# Prevalence and Patterns of Cardiac Arrhythmias in Patients With Chronic Kidney Disease Undergoing Hemodialysis: A Retrospective Study

**DOI:** 10.7759/cureus.100621

**Published:** 2026-01-02

**Authors:** Mohammad Hassan Yousaf, Humzah Abbas, Farrukh Shahzad, Affia Altaf, Saadia Riaz, Maaz Obaid, Dua Rizwan Qureshi

**Affiliations:** 1 Community Medicine, Riphah Islamic International, Rawalpindi, PAK; 2 Cardiology, Mega Medical Complex, Rawalpindi, PAK; 3 Nephrology, Allied Hospital, Faisalabad, PAK; 4 Internal Medicine, Combined Military Hospital, Multan, PAK; 5 Internal Medicine, Sir Ganga Ram Hospital, Lahore, PAK; 6 Emergency Medicine, Lady Reading Hospital, Peshawar, PAK; 7 Internal Medicine, Basic Health Unit, Norai Sharif, PAK

**Keywords:** cardiac arrhythmias, chronic kidney disease, diabetes mellitus, hemodialysis, hypertension

## Abstract

Background

Cardiac arrhythmias are a leading cause of morbidity and mortality among patients with chronic kidney disease (CKD) undergoing maintenance hemodialysis.

Objective

The objective of the study is to determine the prevalence and patterns of cardiac arrhythmias in CKD patients receiving maintenance hemodialysis and to identify clinical and dialysis-related factors associated with arrhythmia occurrence.

Methods

This retrospective cross-sectional study was conducted at Sir Ganga Ram Hospital, Lahore, Pakistan, from March 2022 to March 2025. A total of 155 patients undergoing maintenance hemodialysis were included using non-probability consecutive sampling. Data were extracted from hospital records, dialysis charts, and electrocardiographic (ECG) reports routinely recorded before, during, and after dialysis sessions, as well as 24-hour Holter monitoring reports when clinically indicated. Arrhythmias were categorized as atrial, ventricular, or bradyarrhythmic according to standard ECG criteria, and their timing relative to dialysis sessions (pre-, intra-, and post-dialysis) was documented. Only patients with complete clinical, dialysis, and ECG/Holter data were included.

Results

The mean age of participants was 52.6 ± 12.8 years, with 101 men (65.2%). Hypertension (72.3%), diabetes mellitus (57.4%), and ischemic heart disease (29.7%) were the most common comorbidities. Cardiac arrhythmias were identified in 97 patients (62.6%). Atrial fibrillation was the most frequent sustained arrhythmia (24.5%), followed by ventricular arrhythmias (20.0%) and bradyarrhythmias (10.3%). Arrhythmias were most commonly observed during dialysis (45.4%) and in the post-dialysis period (33.0%).

On multivariate logistic regression analysis, arrhythmia occurrence was independently associated with age > 55 years (odds ratio (OR) = 1.9, 95% confidence interval (CI): 1.1-3.4; p = 0.02), hypertension (OR = 2.1, 95% CI: 1.1-4.0; p = 0.01), diabetes mellitus (OR = 1.8, 95% CI: 1.0-3.3; p = 0.04), ischemic heart disease (OR = 2.4, 95% CI: 1.2-4.9; p = 0.01), dialysis vintage > 5 years (OR = 2.2, 95% CI: 1.2-4.2; p = 0.02), and low-potassium dialysate use (OR = 2.6, 95% CI: 1.3-5.3; p = 0.005).

Conclusion

Cardiac arrhythmias are highly prevalent among patients receiving maintenance hemodialysis, with atrial fibrillation being the most common sustained type. Several patient-related and dialysis-related factors were associated with arrhythmia occurrence; however, these associations should be interpreted in the context of the retrospective, single-center design and clinically indicated rhythm monitoring. Prospective studies with systematic cardiac monitoring are warranted to further clarify causal relationships and preventive strategies.

## Introduction

Chronic kidney disease (CKD) represents a major and growing global public health challenge, affecting an estimated 10%-13% of the adult population worldwide, with a disproportionate burden in low- and middle-income countries (LMICs). Cardiovascular disease remains the leading cause of mortality among patients with advanced CKD receiving maintenance hemodialysis, accounting for nearly 40%-50% of deaths, with cardiac arrhythmias and sudden cardiac death constituting a substantial proportion of these events [1].

Existing epidemiological data suggest that cardiac arrhythmias are highly prevalent in the hemodialysis population, with reported rates ranging from 30% to over 70%, depending on the monitoring modality and population studied [2,3]. Atrial fibrillation (AF) alone affects approximately 15%-30% of patients on long-term hemodialysis-a prevalence two- to threefold higher than that observed in the general population [4]. Ventricular arrhythmias and conduction disturbances further contribute to excess mortality, particularly sudden cardiac death, which remains a leading and often unpredictable outcome in this population [5]. Despite this high burden, much of the existing evidence is derived from registry-based studies and implantable or prolonged Holter monitoring cohorts conducted predominantly in high-income countries, limiting generalizability to LMIC settings.

The pathophysiology underlying arrhythmogenesis in CKD patients undergoing hemodialysis is multifactorial and complex. Structural cardiac remodeling-including left ventricular hypertrophy, myocardial fibrosis, and ischemic heart disease (IHD)-provides a vulnerable arrhythmic substrate, while acute metabolic and hemodynamic shifts during dialysis act as potent triggers [6]. Rapid alterations in serum potassium, calcium, magnesium, and bicarbonate concentrations during dialysis sessions have been shown to influence myocardial repolarization, prolong the QT interval, and increase dispersion of refractoriness, thereby facilitating both atrial and ventricular arrhythmias [7]. Additionally, autonomic dysfunction characterized by sympathetic overactivity and reduced vagal tone further predisposes these patients to electrical instability [8]. Consequently, hemodialysis-although life-sustaining-may paradoxically exacerbate arrhythmia risk.

Among the spectrum of rhythm disturbances, AF remains the most common sustained arrhythmia in dialysis patients and is consistently associated with increased risks of hospitalization, thromboembolic stroke, heart failure, and mortality. Management of AF in this population is particularly challenging due to competing risks of thrombosis and bleeding, underscoring the importance of prevention and early detection [9]. Ventricular arrhythmias, including frequent premature ventricular complexes (PVCs) and non-sustained ventricular tachycardia (NSVT), are also common and are strongly implicated in sudden cardiac death [10]. Bradyarrhythmias, such as sinus node dysfunction and atrioventricular block, though less frequently reported, are increasingly recognized and may necessitate permanent pacing, highlighting their clinical significance [11].

Importantly, emerging evidence indicates that arrhythmic events in hemodialysis patients exhibit distinct temporal patterns related to the dialysis cycle. Studies employing Holter monitoring and implantable loop recorders have demonstrated clustering of ventricular arrhythmias during and immediately after dialysis sessions-periods characterized by rapid fluid shifts, electrolyte changes, and intradialytic hypotension [12]. Conversely, atrial arrhythmias have been observed more frequently during the long interdialytic interval, potentially driven by volume overload and blood pressure variability [13]. Understanding these temporal relationships is clinically relevant, as it provides a rationale for targeted monitoring, risk stratification, and modification of dialysis prescriptions.

Current clinical practice guidelines and expert consensus statements emphasize the importance of identifying modifiable dialysis-related risk factors, such as dialysate potassium and calcium concentration, ultrafiltration rates, and intradialytic hypotension, to mitigate cardiovascular risk in dialysis patients [14,15]. However, data linking these modifiable factors to specific arrhythmia patterns remain limited, particularly in LMIC populations where dialysis practices, resource availability, and patient characteristics may differ substantially from high-income settings.

Against this background, there remains a critical knowledge gap regarding the prevalence, spectrum, and timing of cardiac arrhythmias in hemodialysis patients in South Asia and other LMICs. The present study addresses this gap by providing region-specific data from a large tertiary care center in Pakistan, integrating clinical characteristics, dialysis-related parameters, and arrhythmia timing relative to dialysis sessions. By focusing on routinely available ECG and Holter data in a real-world setting, this study adds novel insight beyond existing registry- and device-based literature and aims to inform clinically actionable strategies for arrhythmia prevention and risk reduction in resource-limited environments.

Objective

The objective of the study is to determine the prevalence and patterns of cardiac arrhythmias in CKD patients receiving maintenance hemodialysis and to identify clinical and dialysis-related factors associated with arrhythmia occurrence.

## Materials and methods

Methodology

This retrospective cross-sectional study was conducted at Sir Ganga Ram Hospital, Lahore, Pakistan, from March 2022 to March 2025. Ethical approval was obtained from the Institutional Review Board/Ethical Review Committee of Sir Ganga Ram Hospital prior to data collection. As this was a retrospective review of anonymized records, the requirement for informed consent was waived. Patient confidentiality was strictly maintained by assigning unique study identification codes, and no personally identifiable information was extracted or reported.

Sampling technique and sample size

A total of 155 patients undergoing maintenance hemodialysis were included. Non-probability consecutive sampling was employed by reviewing all eligible patient records during the study period.

The sample size was determined pragmatically based on the total number of eligible patients available during the study timeframe and is comparable to similar single-center observational studies in the dialysis population. Formal a priori sample size calculation was not performed due to the retrospective nature of the study, which is acknowledged as a limitation.

Inclusion and exclusion criteria

Patients aged ≥18 years with a diagnosis of CKD who had been receiving maintenance hemodialysis for at least three months were included in the study. Eligibility required the availability of complete medical records, dialysis details, and electrocardiographic (ECG) and/or Holter monitoring data. Patients were excluded if they had known congenital or significant valvular heart disease prior to the initiation of dialysis; had permanent pacemakers or implantable cardioverter-defibrillators (ICDs); had acute infection, sepsis, or documented uncontrolled electrolyte imbalance at the time of ECG or Holter recording; or had incomplete or missing essential clinical or dialysis-related information.

Data collection

Data were extracted retrospectively from hospital medical records, dialysis charts, and electronic health records. The collected variables included demographic characteristics (age and gender); clinical variables such as hypertension, diabetes mellitus, IHD, and dialysis vintage; and dialysis-related parameters including session duration, ultrafiltration volume, and dialysate potassium and calcium concentrations.

ECG and Holter monitoring

Standard 12-lead ECGs were reviewed at three time points where available: pre-dialysis, intradialysis, and post-dialysis. Arrhythmia assessment was conducted across multiple dialysis sessions retrospectively, with up to three consecutive dialysis sessions per patient reviewed when ECG data were available.

Twenty-four-hour Holter monitoring was available in a subset of patients, based on clinical indication rather than routine screening. Holter data were used primarily for descriptive analysis of arrhythmia burden and temporal patterns and were not included in the multivariate regression analysis to avoid detection bias between ECG-only and Holter-monitored patients. When multiple ECGs or Holter recordings were available, arrhythmia presence was recorded per patient, and the predominant timing of occurrence was assigned to avoid multiple counting of repeated events.

Operational definitions of arrhythmias

Arrhythmias were operationally defined using standard ECG and Holter criteria: AF was characterized by an irregular rhythm with absent P waves lasting ≥30 s. Atrial flutter was identified by regular atrial activity with saw-tooth flutter waves. Supraventricular tachycardia (SVT) was considered a narrow-complex tachycardia with a sudden onset and termination. PVCs were classified as ≥5 PVCs per minute on ECG or >30 PVCs/hour on Holter. NSVT referred to ≥3 consecutive ventricular beats at a rate > 100 bpm lasting <30 s. Sustained ventricular tachycardia was regarded as ventricular tachycardia lasting ≥30 s or requiring intervention. Bradyarrhythmias were identified as sinus pauses > 3 s or second-/third-degree atrioventricular block.

ECG interpretation and inter-observer reliability

All ECGs and Holter reports were interpreted by consultant cardiologists. In cases where ECG interpretation was ambiguous, a second cardiologist independently reviewed the tracing, and discrepancies were resolved by consensus. Formal inter-observer variability statistics were not calculated, which is acknowledged as a limitation.

Handling of missing data

Only patients with complete key clinical, dialysis, and ECG/Holter data were included in the analysis. Variables with missing values were excluded using complete-case analysis, and no imputation methods were applied, given the retrospective nature and limited proportion of missing data.

Data analysis

Data were analyzed using SPSS version 26.0 (IBM Corp., Armonk, NY, USA). Continuous variables were expressed as mean ± standard deviation (SD), while categorical variables were summarized as frequencies and percentages. Comparisons between groups were performed using an independent Student’s t-test for continuous variables and the Chi-squared (χ²) test for categorical variables. Variables that were clinically relevant and statistically significant on univariate analysis (p < 0.05) were entered into a multivariate logistic regression model to identify independent predictors of arrhythmia. Clinically relevant covariates were retained in the final model regardless of statistical significance to avoid residual confounding. Covariates included age, gender, diabetes mellitus, IHD, dialysis vintage, and selected dialysis-related parameters, chosen based on prior literature, biological plausibility, and observed univariate associations. Age > 55 years and dialysis vintage >5 years were used as dichotomous cut-off values based on the distribution of the study population and thresholds commonly reported in dialysis-related cardiovascular risk studies. Sensitivity analyses using alternative cut-off values demonstrated no meaningful change in the direction or significance of associations. Multicollinearity was assessed before regression modeling using the variance inflation factor (VIF), with values < 2 indicating the absence of significant collinearity. Dialysis-related variables showing substantial collinearity (e.g., ultrafiltration volume, session duration, and intradialytic hypotension) were not simultaneously entered into the final multivariate model to avoid overadjustment. Model performance was evaluated using the Hosmer-Lemeshow goodness-of-fit test and the C-statistic (area under the receiver operating characteristic curve) to assess discrimination. Results were reported as odds ratios (ORs) with 95% confidence intervals (CIs), along with Wald χ² statistics and p-values, with emphasis placed on CIs as measures of uncertainty. A p-value < 0.05 was considered statistically significant.

## Results

Data from 155 patients on maintenance hemodialysis were analyzed (mean age 52.6 ± 12.8 years, range 22-79), including 101 men (65.2%) and 54 women (34.8%). Hypertension was the most common comorbidity, observed in 112 patients (72.3%), followed by diabetes mellitus in 89 (57.4%) and IHD in 46 (29.7%). The mean dialysis vintage was 4.1 ± 2.6 years (Table 1).

**Table 1:** Demographic, Clinical Characteristics, Types, and Timing of Arrhythmias in Hemodialysis Patients (n = 155) *Categories are not mutually exclusive; patients may have more than one arrhythmia or comorbidity. ^†^Arrhythmia prevalence: 62.6% (95% CI: 54.9-70.0) ^‡^Timing percentages calculated only among patients with arrhythmias (n = 97); predominant timing per patient recorded. Data shown as n (%) or mean ± SD. SD: standard deviation; SVT: supraventricular tachycardia; PVCs: premature ventricular complexes; VT: ventricular tachycardia; AV: atrioventricular block; CI: confidence interval

Variable	Category	N	%
Age (years)	Mean ± SD (range)	52.6 ± 12.8 (22-79)	-
Gender	Male	101	65.2
	Female	54	34.8
Comorbidities*	Hypertension	112	72.3
	Diabetes mellitus	89	57.4
	Ischemic heart disease	46	29.7
Dialysis vintage (years)	Mean ± SD	4.1 ± 2.6	-
Arrhythmia prevalence	Any arrhythmia	97	62.6^†^
Types of arrhythmia*	Atrial arrhythmias	50	32.3
	– Atrial fibrillation	38	24.5
	– Atrial flutter/SVT	12	7.7
	Ventricular arrhythmias	31	20
	– PVCs only	21	13.5
	– Non-sustained VT	7	4.5
	– Sustained VT	3	1.9
	Bradyarrhythmias	16	10.3
	– Sinus pauses	9	5.8
	– AV block	7	4.5
Timing of arrhythmia^‡^	Pre-dialysis	21	21.6
	Intradialysis	44	45.4
	Post-dialysis	32	33

Cardiac arrhythmias were identified in 97 patients (62.6%; 95% CI: 54.9-70.0). Patients could exhibit more than one arrhythmia type; therefore, arrhythmia categories were not mutually exclusive, and prevalence for each category was calculated independently. Atrial arrhythmias were the most frequently seen in 50 patients (32.3%; 95% CI: 25.0-39.7), predominantly AF in 38 (24.5%). Ventricular arrhythmias occurred in 31 patients (20.0%), most commonly PVCs, while bradyarrhythmias were observed in 16 patients (10.3%) (Table 1).

Among patients with arrhythmias (n = 97), events were most frequently observed during dialysis (44/97, 45.4%), followed by post-dialysis (32/97, 33.0%) and pre-dialysis (21/97, 21.6%). Timing percentages were calculated only among patients with documented arrhythmias. Timing analyses were descriptive; repeated arrhythmic events within the same patient were not treated as independent observations, and only the predominant timing per patient was recorded to avoid within-subject clustering bias (Table 1).

Patients with arrhythmias were significantly older and had a longer dialysis vintage than those without arrhythmias. Hypertension, diabetes mellitus, and IHD were all more prevalent in the arrhythmia group, whereas gender distribution did not differ significantly (Table 2). Detailed numerical values are presented in the tables; the text highlights only statistically and clinically relevant differences.

**Table 2:** Comparison of Patients With and Without Arrhythmias (n = 155) Continuous data represented as mean ± SD, categorical data as N (%). Continuous variables compared using independent-sample t-test. Categorical variables compared using Chi-squared test. Significance thresholds: p < 0.05 (significant). Percentages reported to one decimal place. SD: standard deviation

Variable	With arrhythmia (n = 97)	Without arrhythmia (n = 58)	Test statistic	p-value
Mean age (years)	55.1 ± 12.2	48.7 ± 13.1	t = 3.00	0.003
Male gender	67 (69.1%)	34 (58.6%)	χ² = 1.54	0.210
Hypertension	78 (80.4%)	34 (58.6%)	χ² = 7.82	0.005
Diabetes mellitus	62 (63.9%)	27 (46.6%)	χ² = 4.02	0.045
Ischemic heart disease	35 (36.1%)	11 (19.0%)	χ² = 5.19	0.023
Dialysis vintage (years)	4.8 ± 2.5	3.2 ± 2.3	t = 3.78	<0.001

Patients with arrhythmias had longer mean dialysis session duration and higher ultrafiltration volumes, both calculated as averages across reviewed dialysis sessions, rather than a single indexed session. Low-potassium dialysate (defined as ≤2.0 mmol/L) and low-calcium dialysate (defined as ≤1.25 mmol/L) were used significantly more often among patients with arrhythmias. Intradialytic hypotension, defined as a drop in systolic blood pressure ≥ 20 mmHg or mean arterial pressure ≥ 10 mmHg requiring intervention, was also more frequent in the arrhythmia group (Table 3). Effect sizes for continuous variables are reflected by mean differences with corresponding p-values, while categorical comparisons are supported by ORs in multivariate analysis.

**Table 3:** Dialysis-Related Factors and Arrhythmia Occurrence (n = 155) Data represented as mean ± SD and N (%). Comparisons performed using independent-sample t-test and Chi-squared test. p < 0.05 = statistically significant. *Values represent average measurements across reviewed dialysis sessions, not a single session. ^†^Low-potassium dialysate defined as ≤2.0 mmol/L. ^‡^Low-calcium dialysate defined as ≤1.25 mmol/L. ^§^Intradialytic hypotension defined as SBP drop ≥ 20 mmHg or MAP drop ≥ 10 mmHg requiring intervention. SD: standard deviation; SBP: systolic blood pressure; MAP: mean arterial pressure

Variable	With arrhythmia (n = 97)	Without arrhythmia (n = 58)	Test statistic	p-value
Mean session duration (hours)*	4.2 ± 0.6	4.0 ± 0.5	t = 2.12	0.036
Mean ultrafiltration volume (L)*	2.8 ± 0.9	2.2 ± 0.8	t = 4.07	<0.001
Low-potassium dialysate^†^	41 (42.3%)	14 (24.1%)	χ² = 4.77	0.029
Low-calcium dialysate^‡^	33 (34.0%)	10 (17.2%)	χ² = 4.35	0.037
Intradialytic hypotension^§^	29 (29.9%)	9 (15.5%)	χ² = 3.98	0.046

Variables entered into the multivariate model were selected based on clinical relevance and univariate significance, including age, comorbidities, dialysis vintage, and dialysate potassium concentration. Dialysis-related variables from Table 3 were evaluated; however, session duration, ultrafiltration volume, low-calcium dialysate use, and intradialytic hypotension were excluded from the final model due to collinearity and overadjustment concerns, particularly their interdependence. Age > 55 years and dialysis vintage > 5 years were dichotomized based on population distribution and prior literature; sensitivity analyses using alternative cut-offs showed no meaningful change in effect direction.

Independent predictors of arrhythmias identified in the multivariate analysis included age greater than 55 years, hypertension, diabetes mellitus, IHD, and a dialysis vintage exceeding five years. Among all variables, the use of a low-potassium dialysate emerged as the strongest independent predictor of arrhythmia occurrence. All clinically relevant variables, including non-significant predictors such as male gender, were retained and reported in the final model (Table 4).

**Table 4:** Binary Logistic Regression Analysis for Predictors of Arrhythmias in Hemodialysis Patients (n = 155) Data represented as odds ratio (OR) ± 95% confidence interval (CI). Analysis performed using binary logistic regression with Wald χ² test. Significance level: p < 0.05 (significant). All clinically relevant variables retained in the model regardless of significance. Dialysis-related variables from Table 3 were assessed but excluded due to collinearity and overadjustment. Model diagnostics: Hosmer-Lemeshow p > 0.05; c-statistic > 0.70. Multicollinearity assessed (VIF < 2 for all predictors). VIF: variance inflation factor

Predictor variable	Adjusted OR	95% CI	Wald χ²	p-value
Age > 55 years	1.9	1.1–3.4	5.26	0.020
Male gender	1.3	0.7–2.5	1.04	0.310
Hypertension	2.1	1.1–4.0	6.32	0.010
Diabetes mellitus	1.8	1.0–3.3	4.19	0.040
Ischemic heart disease	2.4	1.2–4.9	6.58	0.010
Dialysis vintage > 5 years	2.2	1.2–4.2	5.79	0.020
Low-potassium dialysate use	2.6	1.3–5.3	7.87	0.005

Model diagnostics demonstrated good calibration (Hosmer-Lemeshow p > 0.05) and acceptable discrimination (c-statistic > 0.70). Multicollinearity was assessed prior to modeling, with VIF values < 2 for all included variables.

## Discussion

This study highlights the high prevalence of cardiac arrhythmias among patients with CKD receiving maintenance hemodialysis, with nearly two-thirds (97, 62.6%) experiencing at least one arrhythmic episode. This prevalence closely mirrors findings from large international monitoring studies, including the Monitoring in Dialysis (MiD) study, which reported clinically significant arrhythmias in 66% of patients using implantable loop recorders, and the CRASH-ILR study, where arrhythmias were detected in 69% of patients [10,16]. In contrast, lower prevalence rates (35%-45%) have been reported in South Asian cohorts relying primarily on intermittent ECG monitoring, highlighting the influence of monitoring intensity on detection rates [17]. These findings reinforce that arrhythmias represent a major cardiovascular complication in the dialysis population and a significant contributor to sudden cardiac death.

AF was the most common sustained arrhythmia, affecting approximately one-quarter (38, 24.5%) of patients. This estimate is comparable to AF prevalence reported in international dialysis cohorts, ranging from 20% to 27%, and markedly exceeds the 2%-5% prevalence observed in age-matched general populations [8,18]. The clinical significance of AF in dialysis patients is substantial, as it complicates volume control, increases thromboembolic stroke risk, and poses anticoagulation challenges. In our cohort, AF episodes clustered predominantly in the immediate post-dialysis period, a pattern similarly described by Genovesi et al., who reported a 1.8-fold increase in AF occurrence within six hours after dialysis; however, adjustment for dialysis frequency and interdialytic intervals was limited by the retrospective design [19]. Nevertheless, this temporal pattern supports existing mechanistic hypotheses that rapid electrolyte shifts, volume contraction, and autonomic imbalance contribute to arrhythmogenesis.

Ventricular arrhythmias were detected in 31 (20%) patients, with PVCs and NSVT predominating. This prevalence is numerically consistent with implantable loop recorder studies reporting ventricular arrhythmias in 15%-25% of hemodialysis patients [10,16]. Nearly half of these episodes occurred during dialysis sessions, supporting the established relationship between intradialytic hemodynamic stress and arrhythmia occurrence. Prior studies have demonstrated that rapid ultrafiltration, intradialytic hypotension, and low-potassium dialysate increase ventricular arrhythmia risk [6,20]. Our observed association with low-potassium dialysate is directionally consistent with Pun and Middleton, who reported a 40% higher odds of ventricular arrhythmia with dialysate potassium < 2.0 mmol/L [6]. However, this finding should be interpreted cautiously due to potential confounding by indication, as patients perceived to be at higher hyperkalemia risk are more likely to receive lower potassium prescriptions.

Bradyarrhythmias were less frequent (16, 10.3%) but clinically relevant, often presenting with hypotension and pauses during dialysis. Comparable prevalence rates of 8%-15% have been reported in continuous monitoring studies, particularly among patients with autonomic dysfunction and structural heart disease [10,21]. These arrhythmias are likely under-recognized in routine clinical practice but carry important therapeutic implications, including pacemaker consideration in selected cases.

Multivariate analysis demonstrated that age > 55 years, hypertension, diabetes mellitus, IHD, and longer dialysis vintage were independent predictors of arrhythmia occurrence. These findings align with international data showing progressively increased arrhythmia risk with advancing age and dialysis duration, particularly beyond five years [7,19]. Importantly, several dialysis-related parameters were significant on univariate analysis but attenuated after multivariable adjustment, suggesting that part of their apparent effect may be mediated through comorbidity burden and dialysis duration rather than direct causality. These findings align with established cardiovascular risk profiles and underscore the cumulative impact of prolonged dialysis exposure on cardiac electrical instability.

The strong association observed with low-potassium dialysate warrants careful interpretation. Previous studies have shown that while lower dialysate potassium reduces hyperkalemia-related mortality, it may increase arrhythmic risk due to steep potassium gradients [6,22]. This highlights a critical clinical trade-off, underscoring the importance of individualized dialysate prescriptions rather than uniform potassium reduction, particularly in patients with underlying IHD or prolonged dialysis vintage.

Several unmeasured confounders may have influenced the observed associations. Baseline and post-dialysis serum electrolyte levels, medication use (including beta-blockers, antiarrhythmics, and QT-prolonging drugs), and residual renal function were not consistently available for adjustment. Similar limitations have been acknowledged in other retrospective dialysis-arrhythmia studies [16,21]. These factors may partially explain inter-individual variability in arrhythmia risk and should be incorporated into future prospective studies.

The present results have important implications for clinical practice, particularly in resource-limited settings. Routine implantable loop recorders or prolonged Holter monitoring may not be feasible in many LMICs; therefore, risk-stratified approaches using periodic ECGs, targeted Holter monitoring for high-risk patients, and optimization of dialysis prescriptions may represent more pragmatic strategies [17]. Strengthening intradialytic monitoring and early recognition of symptomatic arrhythmias may help mitigate adverse outcomes where advanced monitoring technologies are unavailable.

Several limitations of this study should be acknowledged. First, asymptomatic arrhythmias may have been under-detected due to intermittent ECG monitoring and Holter recordings being available only for a subset of patients. Second, continuous rhythm monitoring was not performed for all participants, which could lead to an underestimation of the true arrhythmia burden. Third, the retrospective design introduces potential data quality limitations and missing variables and precludes causal inference. Fourth, the single-center setting and use of non-probability consecutive sampling may limit external generalizability. Additionally, unmeasured confounders, including baseline electrolyte levels, medication use (e.g., beta-blockers and antiarrhythmics), and residual renal function, were not accounted for in the analysis. Despite these constraints, the study provides valuable, region-specific insight into the prevalence, patterns, and associated factors of arrhythmias in patients receiving maintenance hemodialysis, helping to inform future research and clinical monitoring strategies.

## Conclusions

Cardiac arrhythmias are highly prevalent among patients with CKD undergoing maintenance hemodialysis, affecting nearly two-thirds of the study population. AF was the most common sustained arrhythmia, while ventricular arrhythmias occurred frequently during dialysis sessions, and bradyarrhythmias, although less common, carried potentially important clinical implications. In this single-center, retrospective observational study, older age, hypertension, diabetes mellitus, IHD, longer dialysis vintage, and the use of low-potassium dialysate were found to be significantly associated with arrhythmia occurrence. These associations should be interpreted as correlates rather than causal risk factors.

Given the study’s observational design and single-center setting, the findings should not be overgeneralized beyond similar hemodialysis populations. Nonetheless, the results suggest that closer rhythm surveillance and individualized consideration of dialysis-related parameters, particularly dialysate potassium concentration, may be relevant for patients at higher arrhythmic risk. Importantly, any modification of dialysate composition should be balanced against competing risks, including hyperkalemia, and guided by clinical judgment. Future multicenter prospective studies incorporating systematic continuous rhythm monitoring and interventional evaluation of dialysis prescriptions, particularly dialysate electrolyte composition, are needed to clarify causal relationships and determine whether such strategies can meaningfully influence clinical outcomes.
